# *β-*Synuclein suppresses both the initiation and amplification steps of *α*-synuclein aggregation via competitive binding to surfaces

**DOI:** 10.1038/srep36010

**Published:** 2016-11-03

**Authors:** James W. P. Brown, Alexander K. Buell, Thomas C. T. Michaels, Georg Meisl, Jacqueline Carozza, Patrick Flagmeier, Michele Vendruscolo, Tuomas P. J. Knowles, Christopher M. Dobson, Céline Galvagnion

**Affiliations:** 1 Department of Chemistry, University of Cambridge, Lensfield Road, Cambridge CB2 1EW, UK

## Abstract

*α*-Synuclein is an intrinsically disordered protein that is associated with the pathogenesis of Parkinson’s disease through the processes involved in the formation of amyloid fibrils. *α* and *β*-synuclein are homologous proteins found at comparable levels in presynaptic terminals but *β*-synuclein has a greatly reduced propensity to aggregate and indeed has been found to inhibit *α*-synuclein aggregation. In this paper, we describe how sequence differences between *α*- and *β*-synuclein affect individual microscopic processes in amyloid formation. In particular, we show that *β*-synuclein strongly suppresses both lipid-induced aggregation and secondary nucleation of *α*-synuclein by competing for binding sites at the surfaces of lipid vesicles and fibrils, respectively. These results suggest that *β*-synuclein can act as a natural inhibitor of *α*-synuclein aggregation by reducing both the initiation of its self-assembly and the proliferation of its aggregates.

*α*-synuclein is a 140 residue protein that is intrinsically disordered in solution and is closely involved in the pathogenesis of Parkinson’s disease through its self-assembly to form toxic oligomers and amyloid fibrils^1^,^2^,^3^,^4^,^5^. *α*-synuclein together with another member of the synuclein family, the 134 residue *β*-synuclein, is primarily localised in the terminals of presynaptic neurons^6^. Although the biological functions of these two homologous proteins have not been fully elucidated, *α*-synuclein is thought to play a role in the regulation of synaptic plasticity^7^ and membrane remodelling^8^. In addition, both *α*-synuclein and *β*-synuclein have been shown to interact with lipid vesicles and in each case this interaction induces a high degree of *α*-helical structure^9^,^10^,^11^.

Although both proteins are localised at presynaptic terminals and are expressed at similar levels^12^,^13^, *β*-synuclein has not been implicated in the etiology of Parkinson’s disease but instead has been observed to inhibit the aggregation of *α*-synuclein both *in vitro*^14^,^15^ and *in vivo*^16^,^17^,^18^,^19^,^20^. Altered expression levels of both *α*-synuclein and *β*-synuclein (up-regulated and down-regulated, respectively) have been correlated with disease onset^21^, and counter-regulating the expression of both proteins decreases *α*-synuclein aggregation in mammalian cell cultures, suggesting that *β*-synuclein may act to limit disease progression^21^. Another family member, *γ*-synuclein, is predominantly expressed in sensory neurons and motoneurons of the peripheral nervous system. It has a diffuse distribution throughout axons and the cell body, but has not been associated with the onset or suppression of Parkinson’s disease^22^,^23^.

The N-terminal regions of both *α*-synuclein and *β*-synuclein contain a series of imperfect KTKEGV sequences (7 and 6 repeats, respectively) with a periodicity of 11 residues, a feature that is characteristic of amphipathic helices in apolipoproteins^14^. This region mediates lipid binding^24^,^25^ and is highly conserved between the two proteins; overall, the major sequence difference is a central 11-residue hydrophobic sequence within the non-A*β* component (NAC) region which is present in *α*-synuclein but not in *β*-synuclein (Fig. 1). The C-terminal region of *β*-synuclein shows a much greater degree of sequence divergence from *α*-synuclein than does the N-terminal region, but is similar in being highly acidic and intrinsically disordered even when bound to lipid vesicles^24^,^25^. In *β*-synuclein, however, this region is slightly more proline-rich, and there is evidence that *β*-synuclein populates extended polyproline II conformations in the vicinity of the C-terminus^26^. A range of biophysical studies has shown that *β*-synuclein has a much lower propensity to form amyloid fibrils than does *α*-synuclein^14^,^27^, although insertion of the NAC region into *β*-synuclein does not appreciably alter its aggregation behaviour, even in the presence of sodium dodecyl sulphate (SDS) micelles that considerably accelerate the aggregation of *α*-synuclein^27^,^28^. More recently, methods for predicting the aggregation propensity of polypeptide sequences have been used to design rationally a set of mutations through which residues are swapped to generate variants that aggregate with predictable kinetics^29^.

Despite the fact that *α*-synuclein has a much greater propensity to aggregate than *β*-synuclein, even *α*-synuclein shows remarkable kinetic stability in solution and the initiation of its aggregation requires the involvement of external factors such as suitable interfaces, e.g. air-water boundaries^30^, hydrophobic nanoparticles^31^, or lipid vesicles^32^. In particular, the latter structures are likely to be important for the initiation of aggregation *in vivo*, due to the physiological role of the synucleins as lipid binding proteins. In a recent study, the presence of vesicles composed of negatively charged lipids was found to promote the formation of amyloid fibrils by *α*-synuclein by stimulating the rate of primary nucleation by more than three orders of magnitude at high *α*-synuclein:lipid ratios^32^. Furthermore, it has recently been shown that at mildly acidic pH, autocatalytic secondary nucleation on the surface of existing fibrils exponentially increases the number of fibrils present in solution^33^,^34^. The change in pH from 5.8 to 5.5 was found to increase the rate of secondary nucleation by approximately four orders of magnitude, emphasising the sensitivity of *α*-synuclein aggregation to the solution conditions^34^.

In the work presented here, we have investigated the role of *β*-synuclein on the individual microscopic processes of amyloid fibril formation by *α*-synuclein, i.e. the initial formation of aggregates^32^, their growth, and their autocatalytic amplification^34^. Our data show that *β*-synuclein does not detectably affect *α*-synuclein fibril elongation but inhibits both the lipid-induced aggregation and the autocatalytic amplification of fibrils in the aggregation process, via the competition with *α*-synuclein for binding sites on both lipid vesicles and fibrils, respectively. Crucially, we show that the inhibition does not occur due to interactions between monomeric proteins in solution, but rather by a reduced contact probability at solution interfaces that have now been identified as crucial sites for the initiation of aggregation^32^,^34^. We are therefore able to propose a general mechanistic description for the inhibition of *α*-synuclein aggregation by *β*-synuclein and also to provide new insights into the mechanism of the lipid-induced processes that control the aggregation of *α*-synuclein. Finally, we provide a framework within which we can understand the effects reported *in vivo*^16^,^17^,^18^,^19^ and thus potentially inform on the development of novel *α*-synuclein aggregation inhibitors.

## Results

### Lipid binding and lipid-induced aggregation of *α*- and *β*-synuclein.

**Figure 2:**
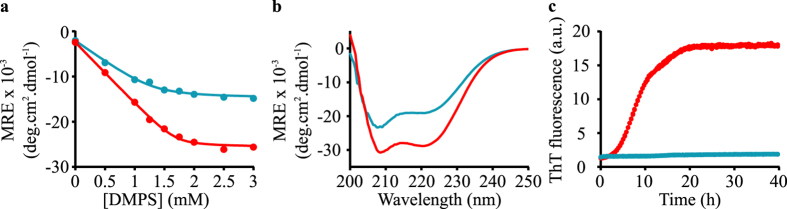
Lipid binding and aggregation properties of *α*-synuclein and *β*-synuclein in the presence of DMPS vesicles. (**a**) Variation of the Mean Residue Ellipticity (MRE) measured at 222 nm when *α*-synuclein (red) and *β*-synuclein (blue) (50 μM) were incubated in the presence of increasing concentrations of DMPS. Both the curves are well described by a one-step binding model (see Methods for details). (**b**) Far UV-CD spectra of *α*-synuclein and *β*-synuclein in the presence of 6 mM DMPS, i.e. under conditions where binding is saturated. (**c**) Change in ThT fluorescence when 200 *μ*M *α*-synuclein (red) or 200 *μ*M *β*-synuclein (blue) was incubated under quiescent conditions in the presence of 350 *μ*M DMPS vesicles. The solution conditions for the CD and fluorescence measurements were 20 mM phosphate buffer, pH 6.5 and 30 °C.

We first investigated the effects of *β*-synuclein on the lipid-induced aggregation of *α*-synuclein. Circular dichroism (CD) was used to study the transition from the intrinsically disordered solution state to the predominantly *α*-helical state of the protein upon binding to vesicles composed of 1,2-dimyristoyl-sn-glycero-3-phospho-L-serine (DMPS) (Fig. 2a,b), and to determine the binding affinity of *β*-synuclein to these vesicles as described previously for *α*-synuclein^32^ (Fig. 2a).

*β*-synuclein was observed to have a lower level of helicity than *α*-synuclein at saturating DMPS concentrations (Fig. 2b, 57 ± 2% helicity for *β*-synuclein compared to 73 ± 4% helicity for *α*-synuclein, see Supplementary Information for details), consistent with previous studies of the binding of the two proteins to detergent micelles and phospholipid vesicles^27^,^35^; this difference can be attributed to the reduced number of imperfect KTKEGV repeats in the N-terminal region of *β*-synuclein as compared to *α*-synuclein. In addition, *β*-synuclein binds to DMPS vesicles with a five-fold lower affinity (*K*_*D,β*_ = 2.57 ± 0.41 *μ*M) than *α*-synuclein (*K*_*D,α*_ = 0.48 ± 0.12 *μ*M) under identical experimental conditions (Fig. 2a), whereas the stoichiometry *L* (defined as the number of lipid molecules in contact with one molecule of synuclein) is similar for both proteins (*L*_*β*_ = 26.5 ± 0.9 and *L*_*α*_ = 33.9 ± 0.5). We then incubated *α*-synuclein and *β*-synuclein separately in the presence of DMPS vesicles (Fig. 2c) and observed that DMPS vesicles initiate *α*-synuclein aggregation, as previously reported^32^, but soluble *β*-synuclein does not form detectable quantities of amyloid species under these conditions during the timescale of this experiment.

**Figure 3:**
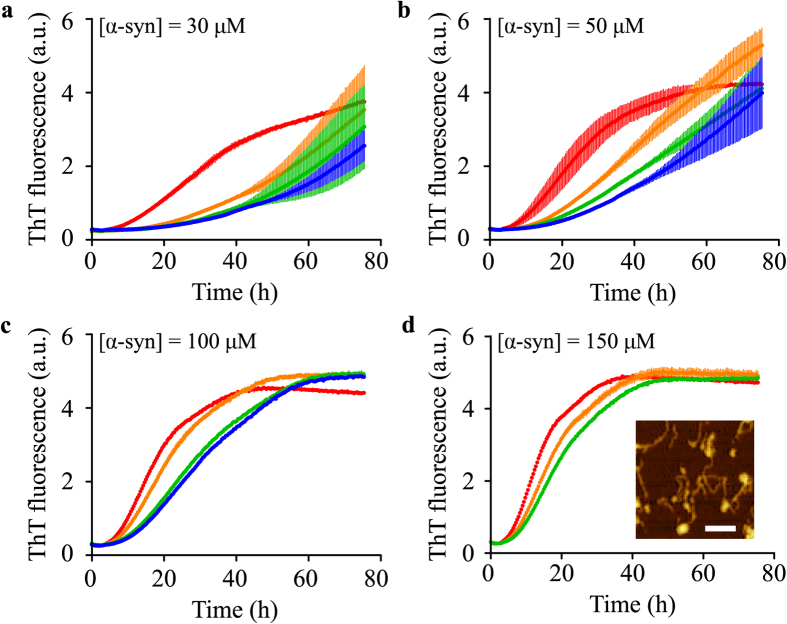
β-synuclein inhibition of *α*-synuclein lipid-induced aggregation. Change in ThT fluorescence when 30 (**a**), 50 (**b**), 100 (**c**) and 150 (**d**) *μ*M *α*-synuclein was incubated under quiescent conditions in the presence of 350 *μ*M DMPS and in the absence (red) and presence of increasing *β*-synuclein concentrations (50 (orange), 100 (green) and 150 (blue) *μ*M). The solution conditions were 20 mM phosphate buffer, pH 6.5 and 30 °C. The thickness of the various lines indicates the standard deviation in triplicate measurements. At longer timescales (see Supplementary Fig. 1) ThT fluorescence reaches the same plateau values. ((**d**), inset) AFM image of fibrils formed in the presence of 150 *μ*M *β*-synuclein (20 *μ*M *α*-synuclein, 350 *μ*M DMPS vesicles, pH 6.5, 30 °C) diluted to 1 *μ*M *α*-synuclein (kinetic data shown in Supplementary Fig. 1). The scale bar is 200 nm.

Having established that *β*-synuclein binds to DMPS vesicles, but that this binding does not induce aggregation, we next studied the potential effect of *β*-synuclein on *α*-synuclein lipid-induced aggregation. For this purpose, we incubated *α*-synuclein and *β*-synuclein at a series of different ratios in the presence of DMPS vesicles under quiescent conditions. As the concentration of *β*-synuclein was increased, the rate of amyloid formation by *α*-synuclein was observed to decrease dramatically (Fig. 3a–d), indicating an inhibitory effect of *β*-synuclein on *α*-synuclein aggregation.

The amyloid fibrils formed in the absence^32^ and the presence of *β*-synuclein however have very similar morphologies, as monitored using atomic force microscopy (AFM) (Fig. 3a, inset) and the same mass of fibrils was formed in each case, with ThT fluorescence reaching the same plateau values (Fig. 3 and Supplementary Fig. 1).

We then investigated whether or not *β*-synuclein was incorporated within *α*-synuclein fibrils using fluorescence spectroscopy and by labelling in turn either *α*-synuclein or *β*-synuclein using the Alexa 568 fluorescent dye (Alexa-568-*α*-synuclein and Alexa-568-*β*-synuclein, respectively) (see Methods for details). At the end of the aggregation reaction, we isolated the fibrils by centrifugation and by comparing the value of the maximum fluorescence intensity at 605 nm of the two types of fibrils, those formed in the presence of Alexa-568-*α*-synuclein and unlabelled *β*-synuclein and those formed in the presence of unlabelled *α*-synuclein and Alexa-568-*β*-synuclein, the upper bound for the percentage of *β*-synuclein incorporation within lipid-induced *α*-synuclein fibrils was found to be 5% (Supplementary Fig. 2c). Moreover, this level of co-localisation of *β*-synuclein in the fibrillar pellet of *α*-synuclein is attributable simply to *β*-synuclein bound to the lipid vesicles present in the pellet rather than to incorporation into the structure of the fibrils. Taken together, these results show that, in the presence of DMPS vesicles, the kinetics of amyloid formation by *α*-synuclein are slowed down by *β*-synuclein without affecting the morphology of the fibrils formed and without a significant degree of co-aggregation of *α*- and *β*-synuclein.

### *β*-Synuclein inhibits *α*-synuclein lipid-induced aggregation via competitive binding to the surface of vesicles

**Figure 4:**
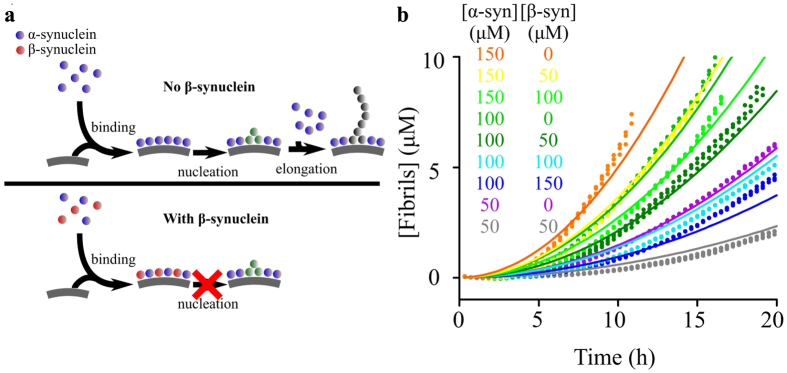
Global analysis of the lipid-induced aggregation of *α*-synuclein in the presence of *β*-synuclein using a competitive lipid binding model. (**a**) Schematic representation of the mechanism of inhibition by *β*-synuclein of the lipid-induced aggregation of *α*-synuclein via competitive binding at the surface of lipid vesicles. (**b**) Change in the concentration of fibrils formed when *α*-synuclein was incubated in the presence of 350 *μ*M DMPS (30 °C, pH 6.5) and increasing concentrations of *β*-synuclein. The protein concentrations used are indicated on the figure. The data were fitted globally using the web-based fitting software AmyloFit^42^ to the kinetic model described in the text (RMSD = 3.61·10^−7^).

In order to explore the mechanism underlying the inhibition by *β*-synuclein of the aggregation of *α*-synuclein in the presence of DMPS vesicles we examined the possibility that the observed inhibition is the result of competitive lipid binding. Although weak interactions between *α*-synuclein and *β*-synuclein in solution have been observed^15^, these are likely to have a negligible effect on the kinetics of the lipid-induced nucleation process investigated here. Indeed, the reaction order, *n*, of the nucleation step with respect to free *α*-synuclein in solution^32^ was found to be close to 0, indicating that the rate determining step of this process is independent of the concentration of free *α*-synuclein. As both *α*-synuclein and *β*-synuclein bind to the vesicles, we considered a mechanism by which *β*-synuclein competes with *α*-synuclein for binding sites at the vesicle surfaces, resulting in the dilution of the *α*-synuclein molecules bound at the surfaces (see Fig. 4a and Methods for details). In our model, a rapid pre-equilibrium between *α*- and *β*-synuclein and a vesicle surface is coupled to the aggregation reaction that is initiated by nucleation on the surface, followed by aggregate growth. We derived an analytical expression based on this model and used it to fit in a global manner the early time points, where the influence of primary nucleation is most marked, of the kinetic traces of *α*-synuclein aggregation in the presence of DMPS and of increasing concentrations of *β*-synuclein. This global analysis describes our entire experimental data very well (Fig. 4b), giving a value for the relevant combined rate constants of nucleation and elongation, *k*_*n*_*k*_+_, of 1.9 ± 0.1·10^−4^ M^−(*n*+1)^ s^−2^, for the apparent reaction order of the nucleation step relative to the concentration of free *α*-synuclein, *n*, of 0.32 ± 0.05, and for the apparent reaction order of the lipid-induced aggregation with respect to the fraction of binding sites on the surface of vesicles occupied by *α*-synuclein, *n*_*b*_, of 5.05 ± 0.03. The values for *k*_*n*_*k*_+_ and *n* are similar (within a factor of two) to those obtained for *α*-synuclein in the absence of *β*-synuclein (*k*_*n*_*k*_+_ of 3.6 ± 0.4·10^−4^ M^−(*n*+1)^ s^−2^, *n* of 0.25 ± 0.01 (Supplementary Fig. 3), indicating that the competition process does not change significantly the underlying reaction mechanism, and that the predominant inhibitory mechanism is a dilution of *α*-synuclein at the membrane surface.

### *β*-Synuclein has no effect on the elongation of *α*-synuclein fibrils

**Figure 5:**
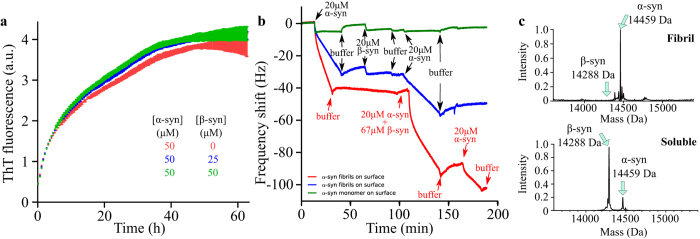
*α*-Synuclein fibril elongation in the presence of *β*-synuclein at pH 6.5, 30 °C. (**a**) Change in the ThT fluorescence signal when solutions of 50 *μ*M monomeric *α*-synuclein were incubated in the presence of 1 *μ*M *α*-synuclein pre-formed seed fibrils in the absence (red) or the presence of 25 *μ*M (blue) or 50 *μ*M (green) monomeric *β*-synuclein. (**b**) QCM measurements at pH 6.5. *α*-synuclein monomers (green) or fibrils (red, blue) were covalently attached to the surface of the QCM sensors. Each trace corresponds to the change in frequency of the corresponding QCM sensor upon incubation with either buffer, *α*-synuclein monomers, *β*-synuclein monomers or a mixture of *α*-synuclein and *β*-synuclein monomers, as indicated in the figure. The red and blue traces show changes in frequencies of QCM sensors with a slightly different surface fibril density, which is responsible for the difference in frequency response upon incubation with a given concentration of monomeric *α*-synuclein. (**c**, top) Mass spectrometric analysis of *α*-synuclein fibrils formed in the presence of *β*-synuclein. The fibrils were first formed by incubating 1 *μ*M *α*-synuclein seed fibrils in the presence 100 *μ*M monomeric *α*-synuclein and 100 *μ*M monomeric *β*-synuclein (kinetic data shown in Supplementary Fig. 4c) and then solubilised after incubation in DMSO prior to mass spectrometric analyses. The data show only one peak, at 14,458.98 ± 0.58 Da, corresponding to the molecular weight of *α*-synuclein, indicating no detectable incorporation of *β*-synuclein within *α*-synuclein fibrils. (**c**, bottom) Mass spectrometric analysis of soluble *β*-synuclein (100 *μ*M) and *α*-synuclein (100 *μ*M) indicating that both proteins can be ionised under the conditions used.

We then investigated the effect of *β*-synuclein on the elongation of *α*-synuclein fibrils. Monomeric *α*-synuclein (50 *μ*M) was incubated under quiescent conditions in the presence of pre-formed *α*-synuclein seed fibrils (1 *μ*M) at pH 6.5 (Fig. 5a and Supplementary Fig. 4) and at increasing concentrations of monomeric *β*-synuclein. The results indicate that *β*-synuclein has no detectable influence on the elongation of *α*-synuclein fibrils (Fig. 5a). Mass spectrometric analysis of the resulting fibrils solubilised in dimethyl sulfoxide (DMSO)^36^ (Fig. 5c, top) showed no signal for *β*-synuclein, although *β*-synuclein can ionise under these conditions (Fig. 5c, bottom), indicating that *β*-synuclein does not detectably incorporate into *α*-synuclein fibrils under these conditions. In addition, analysis of the fibrils formed in the presence of either monomeric Alexa-568-*α*-synuclein or unlabelled monomeric *α*-synuclein and monomeric Alexa-568-*β*-synuclein by fluorescence spectroscopy showed, at the most, a very low degree of incorporation of the latter (<3%, Supplementary Fig. 4b).

We also used microfluidic diffusional sizing^37^ to determine whether or not *β*-synuclein binds to *α*-synuclein fibrils under the conditions of the seeded kinetic experiments. We first measured the effective hydrodynamic radius *R*_*H*_ of monomeric Alexa-568-*β*-synuclein (3 nm) and Alexa-647-*α*-synuclein fibrils (74 nm) alone, and then we incubated monomeric Alexa-568-*β*-synuclein with unlabelled *α*-synuclein fibrils at ratios [*α*-synuclein fibrils]:[*β*-synuclein monomer] (M:M) of 5 and 40. The effective *R*_*H*_ of monomeric *β*-synuclein would increase to that of *α*-synuclein fibrils if monomeric *β*-synuclein interacts tightly with the fibrils. However, we observed only a slight increase in the *R*_*H*_ of monomeric *β*-synuclein from 3 nm to 4.5 nm and 11 nm, at [*α*-synuclein fibrils]:[*β*-synuclein monomer] ratios of 5 and 40, respectively; these results allowed us to estimate the degree of binding to be less than 5% and c.a. 10%, respectively (Supplementary Fig. 5).

Finally, we also investigated both the influence of monomeric *β*-synuclein on *α*-synuclein fibril elongation and the binding between the two species using quartz crystal microbalance (QCM) biosensing. We attached monomeric and fibrillar *α*-synuclein to the surface of a QCM-sensor and incubated the surfaces with monomeric *α*- and *β*-synuclein at pH 6.5 (Fig. 5b). In agreement with the diffusional sizing experiments and the seeded aggregation experiments, we observed only a weak interaction between *β*-synuclein monomers and *α*-synuclein fibrils, and no significant effect of *β*-synuclein on *α*-synuclein fibril elongation at this pH (Fig. 5b). We incubated the surface-bound fibrils simultaneously with *α*-synuclein and an excess of *β*-synuclein, in order to exclude a significant interaction between *β*-synuclein and the *α*-synuclein fibril ends, which could lead to the inhibition of further growth of the fibrils. We observed rapid growth of the fibrils, indicating that *β*-synuclein has no significant affinity for the *α*-synuclein fibril ends. It is interesting to note that the kinetics of the elongation step depends linearly on the concentration of free monomeric *α*-synuclein^34^, however, in our experiments dominated by the elongation of existing fibrils, we found that even an excess of *β*-synuclein had no effect on the kinetics of this process. This finding suggests that weak interactions observed between monomers of *α*- and *β*-synuclein in solution are not responsible for the observed effect on the kinetics. Taken together, these results suggest that *β*-synuclein does not interact strongly with *α*-synuclein fibrils at pH 6.5, a result consistent with its lack of influence on *α*-synuclein fibril elongation.

### *β*-Synuclein inhibits the secondary nucleation of *α*-synuclein aggregation via competitive binding to the fibril surfaces.

**Figure 6:**
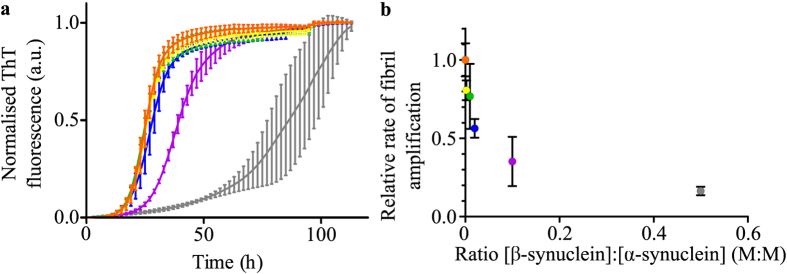
The effect of *β*-synuclein on *α*-synuclein aggregation at mildly acidic pH values. (**a**) Normalised change in the ThT fluorescence signal when 50 nM *α*-synuclein seed fibrils (pH 4.8, 30°C) were incubated under quiescent conditions in the presence of 100 *μ*M monomeric *α*-synuclein in the absence (orange) or presence of increasing concentrations of monomeric *β*-synuclein (0.2 *μ*M (yellow), 1 *μ*M (green), 2 *μ*M (blue), 10 *μ*M (purple) or 50 *μ*M (grey)). (**b**) Quantification of the change in the relative rate of *α*-synuclein fibril amplification with increasing concentration of monomeric *β*-synuclein (See Supplementary Information for details of analysis).

Having established that *β*-synuclein inhibits the lipid-induced aggregation of *α*-synuclein through competition for binding sites on the DMPS vesicles used in this study, we next investigated whether or not a similar effect would also apply to autocatalytic fibril amplification through secondary nucleation at mildly acidic pH values^34^. We therefore incubated 100 *μ*M monomeric *α*-synuclein and very low (50 nM) concentrations of pre-formed *α*-synuclein seed fibrils under quiescent conditions at pH 4.8^34^ in the presence of increasing concentrations of *β*-synuclein. The results reveal that the presence of *β*-synuclein decreases significantly the rate of *α*-synuclein aggregation under these conditions (Fig. 6a,b). In order to be able to disentangle the effects of *β*-synuclein on secondary nucleation and elongation at acidic pH, we also performed experiments at higher seed concentrations, where the aggregation reaction is dominated by elongation. The difference from the seeded kinetics at pH 6.5 is that the elongation rate is approximately one order of magnitude faster, and the higher order assembly (‘flocculation’) of fibrils is strongly enhanced at this pH close to the isoelectric point of the protein^34^. The latter process can affect the elongation rate through the burial of growth competent fibril ends in the higher order aggregates^34^.

The results show, however, as observed at pH 6.5 (Fig. 5), that *β*-synuclein does not alter the kinetics of *α*-synuclein fibril elongation (Supplementary Fig. 6a) indicating that the presence of *β*-synuclein inhibits the process of secondary nucleation but does not alter the elongation rate or higher order assembly behaviour of *α*-synuclein fibrils. Mass spectrometric analysis of the solubilised fibrils formed when *α*-synuclein seed fibrils were incubated in the presence of a mixture of monomeric *α*- and *β*-synuclein at pH 4.8 showed that *β*-synuclein does not incorporate to a detectable extent into *α*-synuclein fibrils under these conditions (Supplementary Fig. 6b). Having thus established that *β*-synuclein influences neither fibril elongation nor higher order assemblies of *α*-synuclein fibrils, we proceeded to analyse quantitatively the inhibitory effect of *β*-synuclein on the autocatalytic amplification of *α*-synuclein fibrils. We found that the apparent secondary nucleation rate constant decreases strongly and in a non-linear manner as the ratio *β*-synuclein:*α*-synuclein increases (Fig. 6b).

**Figure 7:**
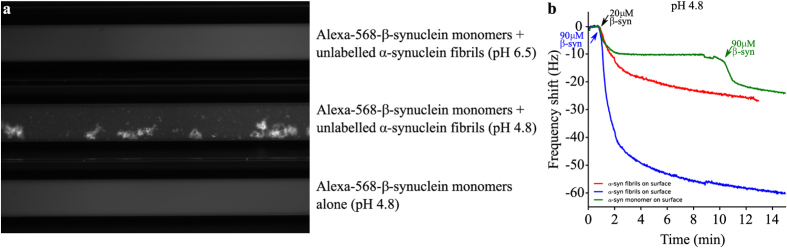
The interaction between *β*-synuclein monomers and *α*-synuclein fibrils at pH 4.8. (**a**) Fluorescence microscopy of monomeric Alexa-568-*β*-synuclein in microcapillaries in the presence of unlabeled *α*-synuclein fibrils at pH 6.5 (top) and pH 4.8 (centre). The bottom capillary shows Alexa-568-*β*-synuclein at pH 4.8 in the absence of *α*-synuclein fibrils. The image in the centre illustrates the binding of *β*-synuclein to *α*-synuclein fibrils at pH 4.8, as well as the higher order assembly of the fibrils. (**b**) Quartz crystal microbalance (QCM) measurements at pH 4.8. *α*-synuclein monomers (green) or fibrils (red, blue) were covalently attached to the surface of the QCM sensors. Each trace corresponds to the change in frequency of the corresponding QCM sensor upon incubation with *β*-synuclein monomers as indicated in the figure.

We then investigated whether or not under these mildly acidic conditions *β*-synuclein can directly interact with the surfaces of *α*-synuclein fibrils, a process which would be able to rationalise the inhibition of secondary nucleation, using both fluorescence microscopy and QCM biosensing measurements. First, we incubated unlabelled *α*-synuclein fibrils with monomeric Alexa-568-*β*-synuclein at pH 4.8 in glass capillaries (Fig. 7a) and we found that *α*-synuclein fibrils (not fluorescent on their own) were fluorescent under these conditions, suggesting the coating of the monomeric Alexa-568-*β*-synuclein to the surface of these assemblies. Incubation of monomeric Alexa-568-*β*-synuclein in the absence of fibrils at pH 4.8 did not result in the formation of fluorescent aggregates (Fig. 7a). Then, we attached monomeric and fibrillar *α*-synuclein to the surface of a QCM-sensor and incubated the surfaces with monomeric *α*- and *β*-synuclein at pH 4.8 (Fig. 7b). We found that *β*-synuclein interacts significantly with surfaces with both attached monomeric and fibrillar *α*-synuclein, with the fibril-coated surfaces displaying a stronger frequency response than the monomer-coated ones (Fig. 7b). It is, however, not straightforward to translate the relative amplitudes into relative affinities or stoichiometries, due to the different degree of surface-coverage by monomer and fibrils, as well as the potential differences in frequency response^38^. Nevertheless, these biosensing experiments confirm that *β*-synuclein strongly interacts with *α*-synuclein fibril surfaces at pH 4.8.

**Figure 8:**
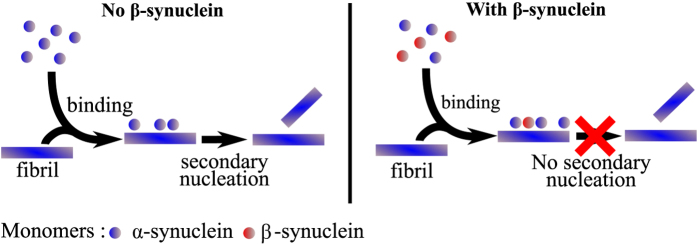
Proposed mechanism of inhibition of *α*-synuclein secondary nucleation by *β*-synuclein via competitive binding to the fibril surface.

Taken together, these results indicate that *β*-synuclein inhibits the secondary nucleation step of *α*-synuclein aggregation by competing with *α*-synuclein monomers for catalytic sites on the surface of *α*-synuclein fibrils (Fig. 8).

## Discussion

Although it has been established that *β*-synuclein can have an inhibitory effect on the aggregation of *α*-synuclein^14^,^15^,^16^,^17^,^18^,^20^, the mechanism by which such inhibition occurs was not previously known but is of very significant biological interest. In earlier work, we have described an experimental and theoretical strategy that has allowed us to dissect the mechanism of aggregation and amyloid formation of *α*-synuclein in great detail^32^,^34^,^39^. In the present study we have applied this approach to investigate how each individual mechanistic step in the overall mechanism of amyloid fibril formation by *α*-synuclein is affected by the presence of *β*-synuclein. The results, consistent with previous findings^14^, indicate that the amino acid sequences of *α*-synuclein and *β*-synuclein are not sufficiently similar to allow a significant degree of cross-aggregation, through which both types of protein molecules would ultimately be incorporated into the ordered *β*-sheet rich structure of amyloid fibrils^40^. On the other hand, the two proteins are sufficiently similar to compete for the same binding sites on surfaces that can induce the formation of *α*-synuclein fibrils, whether the surface is a lipid membrane, the functionally important binding partner of the synucleins, or an *α*-synuclein fibril, whose formation is linked to Parkinson’s disease^4^,^5^. In addition, although recent experimental evidence has shown that *α*-synuclein and *β*-synuclein can form weak dimeric species in solution^15^, our experiments show that this type of interaction appears to have negligible effect on the aggregation behaviour, given the complete lack of inhibition of the elongation reaction by *α*-synuclein. Direct interactions between *α*- and *β*-synuclein may still be relevant, however if they occur between their lipid or fibril-bound forms.

The present study highlights the important observation that indirect mechanisms can play a very significant role in modulating pathways leading to amyloid formation. Thus, for example, for secondary nucleation, an inhibitor acting on the fibril surface (as seen for a molecular chaperone domain, Brichos, in the context of A*β*42 aggregation^41^) can be at least as effective as one binding directly to *α*-synuclein. Moreover, in the case of lipid-induced aggregation, the prevention of a high local concentration of *α*-synuclein on the surfaces of membranes can be highly effective in inhibiting such behaviour, as reflected by the high apparent reaction order of the overall aggregation process with respect to the fraction of binding sites on the surface of vesicles occupied by *α*-synuclein (~5). However, we would like to emphasize that it is not straightforward to convert this reaction order, which corresponds to the combined reaction order of nucleation and elongation, into a nucleus size^42^. As the focus of the major origins of toxicity has shifted in recent years from fibrillar forms of amyloidogenic proteins to smaller, more soluble and more mobile oligomeric species^2^, interfering with the interaction of soluble *α*-synuclein with the surfaces of *α*-synuclein fibrils, a process that can lead to the generation of new oligomeric species through secondary nucleation^34^,^43^, could effectively suppress the proliferation of amyloid fibrils, and therefore be a potential therapeutic strategy.

Previous work using a transgenic mouse model has shown that viral delivery of the *β*-synuclein gene reduces both membrane translocation and aggregation of *α*-synuclein^17^. In addition peptides derived from *β*-synuclein appear to be neuroprotective in tissue culture models of *α*-synuclein pathology, with residues 1–15 of *β*-synuclein (which are involved in lipid binding^27^) being particularly important^19^. Our results indicate that the mode of action of *β*-synuclein in these studies is likely to be, at least in part, a result of competitive binding to lipid vesicles that we have demonstrated in the present study to have a very significant influence *in vitro* on the kinetics of *α*-synuclein aggregation.

We have therefore been able to provide a description of the effect of *β*-synuclein on *α*-synuclein aggregation that rationalises the selective interference of *β*-synuclein with the dominant surface-induced fibril production processes. Furthermore, these systematic experimental studies on the effects of *β*-synuclein have allowed us to gain additional insights into the complex aggregation mechanism of *α*-synuclein. Moreover, the results of this *in vitro* study reveal that the ability of *β*-synuclein to inhibit the aggregation of *α*-synuclein, at least under the conditions studied here, is a consequence of competitive binding to the surfaces that otherwise trigger the process of fibril formation. The presence of *β*-synuclein could, therefore, contribute to the suppression of both the initiation of aggregation and the proliferation of aggregates *in vivo* and hence acts as a natural inhibitor of both the onset and spreading of neurodegeneration in disorders such as Parkinson’s disease.

## Methods

### Materials

1,2-Dimyristoyl-sn-glycero-3-phospho-L-serine (sodium salt; DMPS) was purchased from Avanti Polar Lipids (Alabama, USA). Sodium phosphate monobasic (NaH_2_PO_4_, BioPerformance Certified, ≥99.0%), sodium phosphate dibasic (Na_2_HPO_4_, ReagentPlus, ≥99.0%), sodium azide (NaN_3_, ReagentPlus, ≥99.5%) and carbon black nanopowder were purchased from Sigma Aldrich (Poole, UK). Thioflavin T UltraPure Grade (ThT, ≥95%) was purchased from Eurogentec (Southampton, UK) and polydimethylsiloxane (PDMS) (Sylgard 184 kit) from Dow Corning (Midland, Michigan). Alexa Fluor 568 NHS-ester and Alexa Fluor 647 maleimide were purchased from Life Technologies (Carlsbad, California).

### Protein and lipid preparation

Wild-type *α*-synuclein and *β*-synuclein were expressed and purified as described previously for *α*-synuclein^44^ with an additional final size-exclusion chromatography step in which the proteins were eluted in 20 mM phosphate buffer (NaH_2_PO_4_/Na_2_HPO_4_), pH 6.5, 0.01% NaN_3_). Seed fibrils (preformed fibrils used for seeding the aggregation of *α*-synuclein) were formed by incubating 500 *μ*l solutions of *α*-synuclein at a concentration of 300 *μ*M in 20 mM phosphate buffer at pH 6.5 for 72 h at 45 °C under stirring conditions with a Teflon bar. At 24 h intervals, the solutions of fibrils were sonicated for 3 × 10 s using a probe sonicator (Bandelin, Sonopuls HD 2070, Berlin, Germany) (10% maximum power and 10% cycles). After 72 h, the fibril solutions were divided into 50 *μ*l aliquots, flash frozen with liquid N_2_ and stored at −20 °C until required. For aggregation experiments in the presence of seed fibrils, the solutions were diluted to 5–50 *μ*M in water and sonicated for a further 3 × 10 s using a probe sonicator (10% maximum power and 10% cycles) just before use. DMPS vesicles were prepared as described previously^32^. Alexa-647-*α*-synuclein fibrils were formed from N122C *α*-synuclein labelled with Alexa Fluor 647 maleimide as previously described^45^.

### Circular dichroism spectroscopy

CD spectroscopy was carried out using 50 *μ*M *α*-synuclein or *β*-synuclein in the presence of increasing concentrations of DMPS in 20 mM phosphate buffer, pH 6.5, 0.01% NaN_3_. Far-UV CD spectra were recorded on a JASCO J-810 spectropolarimeter (JASCO, Easton, Maryland) equipped with a Peltier thermally controlled cuvette holder at 30 °C. Quartz cuvettes with path lengths of 1 mm were used and the CD signal intensity at 222 nm was obtained by averaging 20 data sets, each acquired for 1 s. For each protein sample, the CD signal of the buffer used to solubilise the protein was recorded and subtracted from that of the protein. The CD data were analysed as described previously^32^ for *α*-synuclein and estimates of the proportions of different forms of secondary structure were obtained using CDPro software (CONTIN/LL, reference set 8).

### Microfluidics

Microfluidic diffusion devices were fabricated according to previously published protocols^37^,^46^. Devices were cast using polydimethylsiloxane (PDMS) from a silicon wafer master imprinted with 25 *μ*m high channels. Carbon black nanopowder was added to the PDMS to minimise fluorescent scattering during measurements. Devices were bonded to glass slides and oxidised with plasma treatment (Electronic Diener Femto plasma bonder, Diener Electronic, Ebhausen, Germany). A needle (25 gauge, Neolus Terumo, Leuven, Belgium) and tubing was fitted to a 500 *μ*L glass syringe (Hamilton, Sigma Aldrich) containing buffer to fill each device. Samples were incubated for 15 min prior to loading. Gel-loading tips containing sample and buffer (including 0.1% Tween-20 to eliminate adhesion to the sides of the channels; note that Tween-20 was not added to the sample itself) were inserted into inlets and withdrawn via a syringe pump (Cetoni neMESYS, Korbussen, Germany) with flow rates from 40–150 *μ*L/h. Images of the 12 diffusion positions along the length of the diffusion channel were taken on an inverted epifluorescence microscope (Axio Observer D1, Zeiss, Oberkochen, Germany) using a LED light source (Cairn Research, Faversham, Kent) with 49004 and 49009 filter cubes (Chroma Technology Corporation, Bellows Falls, Vermont). A CCD camera (Evolve512, Photometrics, Tucson, Arizona) was used to acquire the images, with 2 s exposure time. The details of the analysis is described in the Supplementary Information.

### Fluorescence microscopy in glass microcapillaries

Experiments were performed in square microcapillaries fabricated from borosilicate glass with inner dimensions of 200 *μ*m × 200 *μ*m and a wall thickness of 100 *μ*m (CM Scientific, Silsden, UK). The samples that contained *α*-synuclein fibrils were prepared by mixing 2.5 *μ*l fibrils at 400 *μ*M (produced^34^ at pH 6.5, not sonicated) with 1 *μ*l of 270 *μ*M *β*-synuclein solution (fluorescently labeled with Alexa 568 NHS ester, see Supplementary Information for details of labeling protocol) and 100 *μ*l 20 mM phosphate buffer (pH 6.5, or pH 4.8; the latter corresponds to a solution of pure NaH_2_PO_4_ which has virtually no buffering capacity. However, due to the strong dilution, the final pH will be close to 5). For the samples without fibrils, the 2.5 *μ*l aliquots of fibril solution were omitted, otherwise the conditons were the same as in the samples with fibrils. The capillaries were filled by dipping into the sample solution (through capillary action) and sealed with sealing wax (Hawksley, Lancing, UK). Images of the capillaries were taken with an inverted epifluorescence microscope (see above) using a LED light source with a 49004 filter cube.

### Quartz crystal microbalance measurements

QCM experiments were performed with a E4 QCM-D instrument (Q-Sense, Biolin Scientific, Stockholm, Sweden), using gold-coated QSX 301 sensors. The protocols for the attachment of monomeric and fibrillar *α*-synuclein closely followed methods presented previously^47^. See Supplementary Information for further details.

### Aggregation kinetics

For aggregation kinetics experiments, ThT fluorescence intensity was recorded in 96-well low-binding clear-bottomed plates (Corning, Corning, New York) using Fluorostar Optima, Polarstar Omega and Clariostar (BMG Labtech, Aylesbury, UK) fluorescence plate-readers in bottom-reading mode. All experiments were performed under quiescent conditions with a ThT concentration of 50 *μ*M, and the data presented are either individual traces or averages and standard deviations of 3 repeats. At the end of each aggregation experiment, the fibril concentration was determined by ultracentrifugation of the sample for 30 min at 90 k rpm and determining the remaining free monomer concentration in the supernatant by measuring absorbance at a wavelength of 280 nm using an extinction coefficient of 5,960 M^−1^cm^−1^, correcting for ThT absorbance as described previously^32^. See Supplementary Information for details of analysis and determination of the degree of co-aggregation.

### Atomic force microscopy

Samples were taken at the end-point of the aggregation reaction and diluted in dH_2_O to 1 *μ*M total protein concentration. 10 *μ*l of these were pipetted onto freshly cleaved mica (Agar Scientific, Stansted, UK) on glass slides and left to dry. The samples were imaged with a Nanowizard II atomic force microscope (JPK Instruments, Waterbeach, UK) in intermittent contact mode in air. NSC 36 cantilevers were used (Mikromasch, Tallinn, Estonia) with resonant frequencies between 70 kHz and 150 kHz.

## Additional Information

**How to cite this article**: Brown, J. W. P. *et al. β*-Synuclein suppresses both the initiation and amplification steps of *α*-synuclein aggregation via competitive binding to surfaces. *Sci. Rep.* **6**, 36010; doi: 10.1038/srep36010 (2016).

**Publisher’s note**: Springer Nature remains neutral with regard to jurisdictional claims in published maps and institutional affiliations.

## Supplementary Material

Supplementary Information

## Figures and Tables

**Figure 1:**
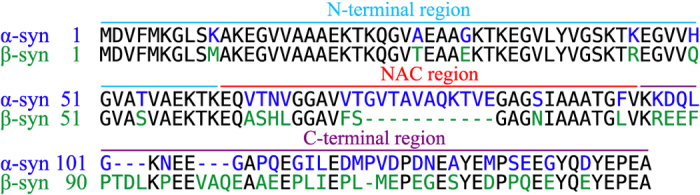
Sequence alignment of *α*-synuclein and *β*-synuclein showing the three distinct regions of the protein. Identical residues are coloured black and different residues are coloured in either blue or green depending on whether they belong to *α* or *β*-synuclein, respectively. The sequences were aligned using EMBOSS - Needle (http://www.ebi.ac.uk/Tools/ psa/emboss_needle/). The sequence identity for these two proteins is 61.6%.
