# Supplementary material for: β-Synuclein suppresses both the initiation and amplification steps of α-synuclein aggregation via competitive binding to surfaces
Source: Sci Rep. 2016 Nov 3;6:36010. doi: 10.1038/srep36010 (PMC5093550; doi:10.1038/srep36010)
Supplement: Supplementary Information [file srep36010-s1.pdf]

Supplementary Information: β-Synuclein suppresses both the initiation and amplification steps of α-synuclein aggregation via competitive binding to surfaces

James W. P. Brown[1], Alexander K. Buell[1,2], Thomas C.T. Michaels[1], Georg Meisl[1], Jacqueline Carozza[1], Patrick Flagmeier[1], Michele Vendruscolo[1], Tuomas P.J. Knowles[1], Christopher M. Dobson[1*], Céline Galvagnion[1,2,3*]
[1] Department of Chemistry, University of Cambridge, Lensfield Road, Cambridge CB2 1EW, UK
[2] Present address: Institute of Physical Biology, University of Düsseldorf, Universitätsstr.1, 40225 Düsseldorf, Germany
[3] Present address: German Center for Neurodegenerative Diseases (DZNE), Ludwig-Erhard-Allee 2, 53175 Bonn, Germany
Correspondence should be addressed to C.G. (celine.galvagnion@dzne.de) and C.M.D. (cmd44@cam.ac.uk)

Supplementary Methods

Calculation of the fraction of binding sites on the surface of vesicles occupied by α-synuclein

α-Synuclein and β-synuclein bind to DMPS vesicles with dissociation constants, $K_{D,\alpha}$ and $K_{D,\beta}$, respectively, and stoichiometries, L_α and L_β, respectively. Thus, for increasing β-synuclein:α-synuclein ratios, the fraction of protein binding sites on the surfaces of the vesicles occupied by α-synuclein molecules (θ_α) will decrease as β-synuclein competes for the same protein binding sites. We therefore used a competitive binding model, which makes the assumption that α-synuclein and β-synuclein bind independently to DMPS vesicles, to determine θ_α for any β-synuclein:α-synuclein ratio:

$$\theta_\alpha = \frac{[\alpha_b]L_\alpha}{[DMPS]} \tag{S1}$$

where $[\alpha_b]$ is the concentration of α-synuclein bound to the DMPS vesicles, $[DMPS]$ is the total DMPS concentration and $\frac{[DMPS]}{L_\alpha}$ is the concentration of protein binding sites at the surfaces of the vesicles.

We then expressed the total concentrations of DMPS and α- and β-synuclein as a function of the binding constants $K_{D,\alpha}$ and $K_{D,\beta}$ of α- and β-synuclein for the DMPS vesicles using the relationships:

$$[DMPS] = [DMPS_f] + L_\alpha[\alpha_b] + L_\beta[\beta_b] \tag{S2}$$

$$[\alpha] = [\alpha_f] + [\alpha_b] \tag{S3}$$

$$[\beta] = [\beta_f] + [\beta_b] \tag{S4}$$

$$K_{D,\alpha} = \frac{[\alpha_f][DMPS_f]}{L_\alpha[\alpha_b]} \tag{S5}$$

$$K_{D,\beta} = \frac{[\beta_f][DMPS_f]}{L_\beta[\beta_b]}, \tag{S6}$$

where $[DMPS_f]$, $[\alpha_f]$ and $[\beta_f]$ are the concentrations of free DMPS, free α-synuclein and free β-synuclein, respectively, $[\beta_b]$ corresponds to the concentration of β-synuclein bound to DMPS and $[\alpha]$ and $[\beta]$ are the total concentrations of α- and β-synuclein, respectively. By combining equations S2-S6 we can derive an expression for $[\alpha_b]$ in terms of $K_{D,\alpha}$, $K_{D,\beta}$, L_α, L_β, $[\alpha]$, $[\beta]$ and $[DMPS]$. This relationship is obtained as the solution to the following cubic equation:

$$K_{D,\alpha} = \frac{([DMPS] - L_\beta[\beta_b] - L_\alpha[\alpha_b])([\alpha] - [\alpha_b])}{[\alpha_b]L_\alpha} \tag{S7}$$

where

$$[\beta_b] = \frac{[DMPS] - [\alpha_b]L_\alpha + K_{D,\beta}L_\beta + L_\beta[\beta] - \sqrt{X}}{2L_\beta} \tag{S8}$$

and

$$X = 4L_\beta([\alpha_b]L_\alpha[\beta] - [DMPS][\beta]) + ([DMPS] - [\alpha_b]L_\alpha + K_{D,\beta}L_\beta + L_\beta[\beta])^2$$

The explicit expression is not shown here due to its length. The influence of different concentrations of β-synuclein on the binding of α-synuclein can be simulated using this model and is shown in Supplementary Figure 6.

Microfluidics - Data analysis

The 12 images taken along the diffusion channel were processed into a set of lateral scan profiles, which were then fitted to a set of simulated basis functions. These diffusion profiles contain information both about the spatial diffusion (along the channel width) and temporal evolution (along the channel length), which allows deconvolution of the experimental profiles and measurement of the diffusion coefficients for individual components of a mixture. A maximum entropy basin hopping algorithm with a Broyden Fletcher Goldfarb Shannon (BFGS) minimisation protocol was used to find the linear combination of simulated radii that gives the lowest residuals. This analysis yields the average radius of the sample, as well as the fractions of individual species if the sample is polydisperse[1, 2].

Quartz Crystal Microbalance measurements

The QCM experiments were performed with a E4 QCM-D instrument (Q-Sense, Biolin Scientific, Stockholm, Sweden), using gold-coated QSX 301 sensors. The sensors were cleaned immediately prior to use with a hot (80°C) mixture of water/30% H_2O_2/conc. NH_3 solution (5/1/1) for 30 min, followed by rinsing with water, drying, and UV/O_3 treatment. The protocols for the attachment of monomeric and fibrillar α-synuclein closely followed methods presented previously [3]. Both fibrillar and monomeric α-synuclein samples were prepared in PBS buffer at 20μM. The fibrils were sonicated for 4 min at 20% amplitude on a 500 W ultrasonic homogenizer (Cole Parmer, Hanwell, UK) with 3 s pulses and 3 s breaks. 2-Iminithiolane (Traut's reagent) was added to give a final concentration of 1 mg/ml to the protein samples, which were incubated for 5 min and then 80 μl aliquots of the chemically activated protein solution were put onto each of the freshly cleaned gold-coated sensors for 1h in a humid atmosphere, to prevent drying. Then the sensor surfaces were rinsed with PBS buffer and incubed for 30 min with a 1% (v/v) solution of mPEG-thiol (Polypure, Oslo, Norway) in PBS. Then the sensors were rinsed with water and introduced into the microbalance. The QCM flow cells were filled with phosphate buffer at pH 6.5 and the temperature was set to 37°C. The measurements were started once a stable frequency baseline had been established. The solutions of α-synuclein and β-synuclein for the interaction studies were introduced via a peristaltic pump at a flow rate of 150 μl/min. For each sample, 300 μl aliquots were introduced and the pump stopped during the measurements.

Aggregation kinetics

Data acquisition

Lipid-induced aggregation
Lipid-induced aggregation experiments were performed under quiescent conditions at 30 °C, pH 6.5 in the presence of 350 μM DMPS vesicles and increasing concentrations of α-synuclein and β-synuclein (0 - 200 μM), as described in the Results section. At the end of each aggregation experiment, the fibril concentration was determined by ultracentrifugation of the sample for 30 min at 90 k rpm and determining the remaining free monomer concentration in the supernatant by measuring absorbance at a wavelength of 280 nm using an extinction coefficient of 5,960 $M^{-1}cm^{-1}$, correcting for ThT absorbance as described previously[4]. For Alexa-568-α-synuclein, a correction for absorbance by the Alexa-568 dye was also performed, according to the manufacturer's protocol (Life Technologies, Carlsbad, California). By assuming that the ThT signal was proportional to the fibril mass, the change in mass over the time course of the experiment could then be calculated.

Seeded aggregation
Experiments using α-synuclein seed fibrils were performed under quiescent conditions at 30 °C under two different solution conditions in order to study selectively either their elongation or autocatalytic amplification[5]. Fibril elongation was studied when monomeric α-synuclein (10-100 μM) was incubated in the presence of micro-molar concentrations of seed fibrils, whereas fibril amplification behaviour was studied when monomeric α-synuclein (10-100 μM) was incubated in the presence of nano-molar concentrations of seed fibrils below pH 5.8[5]. We followed the change in ThT fluorescence (50 μM) in the presence of either (i) 5 μM α-synuclein seed fibrils at pH 4.8 and 6.5 (20 mM phosphate

buffer, 0.01% NaN_3) in the presence of 100 μM monomeric α-synuclein and increasing concentrations of monomeric β-synuclein (0-50 μM) or (ii) 50 nM α-synuclein seed fibrils at pH 4.8 (20 mM phosphate buffer, 0.01% NaN_3) in the presence of 100 μM monomeric α-synuclein and increasing concentrations of monomeric β-synuclein (0-50 μM).

Determination of the degree of co-aggregation of α- and β-synuclein

In order to detect the relative quantities of α-synuclein and β-synuclein incorporated within the fibrils formed in the presence of DMPS, α-synuclein and β-synuclein were each labelled at their primary amine groups with Alexa Fluor 568 (Alexa-568) by NHS ester conjugation according to the manufacturer's protocol (Life Technologies, Carlsbad, California). Each protein was then purified by a single size exclusion chromatography (Superdex 200 Increase 10/300 GL, GE Healthcare, Buckinghamshire, UK) step in which labelled monomeric protein was eluted in 20 mM phosphate buffer, pH 6.5, 0.01% NaN_3. The efficiency of labelling was determined using UV absorbance at 280 nm and 577 nm to be c.a. 10 %. Fibrils were formed by incubating 100 μM monomeric Alexa-568-β-synuclein in the presence of an equal concentration of unlabelled monomeric α-synuclein and either DMPS vesicles or α-synuclein seed fibrils. When the ThT signal reached the plateau phase, the reaction mixtures were ultracentrifuged (90 krpm for 30 min) and the pellets washed with the reaction buffer (20 mM phosphate buffer, pH 6.5, 0.01% NaN_3) in order to isolate the resulting fibrils and to wash off any monomeric α-synuclein and β-synuclein. This step was carried out twice before measuring the fluorescence spectrum of the resuspended fibrils from 580 and 680 nm.

Labelling reduced the lipid binding affinities and slowed down the kinetics of lipid-induced α-synuclein aggregation, effects likely to be due to a perturbation of the α-helical region upon addition of the negatively charged dye at the N-terminus, but did not change the mass of fibrils formed (Supplementary Figure 2). We compared the fluorescence intensity at 600 nm of fibrils formed when α-synuclein seed fibrils were incubated in the presence of unlabelled monomeric α-synuclein and Alexa-568-β-synuclein ($I_{600\mathrm{nm,Alexa-568-}\beta\mathrm{-syn}}$) to that of fibrils formed when α-synuclein seed fibrils were incubated in the presence of monomeric Alexa-568-α-synuclein and unlabelled β-synuclein ($I_{600\mathrm{nm,Alexa-568-}\alpha\mathrm{-syn}}$). The ratio of the two intensities gives the upper bound of the level of incorporation of β-synuclein within α-synuclein fibrils.

Theoretical modeling

α-synuclein aggregation in the presence of lipid vesicles

A full kinetic model for α-synuclein aggregation in the presence of lipid vesicles involves the simultaneous action of protein binding to vesicles, surface primary nucleation and fibril elongation. In the limit of fast binding of α- and β-synuclein onto the surface of vesicles, the resulting set of kinetic equations can be solved by analysis through the method of matched asymptotics. The resulting analytical solution evolves through two main reaction stages. In the first stage (boundary layer) a quasi-equilibrium between bound and free α- and β-synuclein is established, leading to Eqs. S5 and S6. In the second stage (slow manifold) α-synuclein molecules bound to vesicles nucleate and generate aggregates that are capable of further growth. Under these circumstances, the rates of the nucleation and growth steps depend on θ_α to powers ν and ϵ, respectively. The fundamental kinetic equations describing this slow stage of the reaction are therefore given by:

$$\frac{d[\alpha_f]}{dt} = -k_+ \frac{[\alpha_f]\theta_\alpha^\epsilon}{1+\frac{[\alpha_f]}{K_M}}[P] \tag{S9}$$

$$\frac{d[P]}{dt} = k_n[\alpha_f]^n \theta_\alpha^\nu \frac{[DMPS]}{L_\alpha}, \tag{S10}$$

where $[P]$ is the number concentration of aggregates, k_+, k_n are the rate constants for elongation and primary nucleation in the presence of DMPS, respectively, n is the reaction order of the nucleation step relative to free monomeric α-synuclein, ν and ϵ are the reaction orders of the nucleation and elongation steps, respectively, and K_M is the Michaelis-Menten constant describing saturating filament elongation. The early time behaviour of the solution to Eqs. S9 and S10 ($[\alpha_f](t) = [\alpha_f](0)$) yields a t^2-behaviour for the mass concentration $[M]$ of aggregated protein:

$$[M_{\mathrm{early}}] = \frac{k_+k_n[\alpha_f]^{n+1}\theta_\alpha^{n_b}}{\left(1+\frac{[\alpha_f]}{K_M}\right)} \cdot \frac{[DMPS]}{L_\alpha} \cdot \frac{t^2}{2}. \tag{S11}$$

where $n_b = \nu + \epsilon$ is the reaction order of lipid induced aggregation with respect to θ_α. A global fit of aggregation data at early time points was performed using this model and the web-based software AmyloFit[6]. It is noteworthy, that the analytical solution used previously to describe the lipid-induced aggregation data [4] corresponds to Eq S11 under conditions of full lipid coverage by one species (α-synuclein) corresponding to $\theta_\alpha = 1$.

Aggregation of α-synuclein at pH 4.8

Fibril amplification processes (processes involving a change in the number of fibrils) of α-synuclein aggregation are greatly enhanced at mildly acidic pH[5]. They are most readily observed in the presence of nanomolar seed fibril concentrations and micromolar monomeric α-synuclein concentrations[5].

We begin with the linear polymerization model[7]:

$$\frac{dm(t)}{dt} = -\frac{dM(t)}{dt} = -2k_+ P(t)m(t) \tag{S12}$$

and take the derivative of $\frac{dM(t)}{dt}$,

$$\frac{d^2M(t)}{dt^2} = 2k_+ \left(\frac{dm(t)}{dt} P(t) + \frac{dP(t)}{dt} m(t) \right) \tag{S13}$$

which allows us to formulate an equation for the rate of fibril amplification, $\frac{dP(t)}{dt}$:

$$\frac{dP(t)}{dt} = \frac{1}{2k_+ m(t)} \left(\frac{d^2M(t)}{dt^2} - 2k_+ \frac{dm(t)}{dt} P(t) \right) \tag{S14}$$

Where k_+ is the fibril elongation rate constant, $m(t)$ the soluble monomer concentration, $M(t)$ is the mass concentration of fibrils and $P(t)$ the number concentration of fibrils. At early times in the aggregation reaction, the monomer concentration and the fibril number concentration can be assumed to be constant, hence $m(t) = m(0)$ and $P(t) = P(0)$. At the half-time of the reaction, $\frac{d^2M(t)}{dt^2}$ is small for sigmoidal curves; we therefore assume that $\frac{d^2M(t)}{dt^2} \approx 0$, resulting in:

$$\left.\frac{dP(t)}{dt}\right|_{t=t_{1/2}} = -\frac{1}{m(t=t_{1/2})} P(t=t_{1/2}) \left.\frac{dm(t)}{dt}\right|_{t=t_{1/2}} \tag{S15}$$

Since $m(t=t_{1/2}) = 0.5m(0)$, $P(t=t_{1/2})$ can be substituted with $-\frac{1}{0.5m(0)k_+} \left.\frac{dm(t)}{dt}\right|_{t=t_{1/2}}$, which gives:

$$\left.\frac{dP(t)}{dt}\right|_{t=t_{1/2}} = \left(\frac{1}{m(0)} \left.\frac{dm(t)}{dt}\right|_{t=t_{1/2}} \right)^2 \frac{4}{k_+} \tag{S16}$$

Our results show that β-synuclein does not perturb the elongation of α-synuclein fibrils under the solution conditions where secondary nucleation can be studied, i.e. the presence of β-synuclein does not appear to affect k_+. In the experiments designed to probe the effect of β-synuclein on the secondary nucleation of α-synuclein (see details at the beginning of this section), the α-synuclein concentration, $m(0)$, was kept constant, so for the purposes of this analysis the only variable in equation S16 is $\left.\frac{dm(t)}{dt}\right|_{t=t_{1/2}}$. Therefore:

$$\left.\frac{dP(t)}{dt}\right|_{t=t_{1/2}} \propto \left(\left.\frac{dm(t)}{dt}\right|_{t=t_{1/2}} \right)^2 \tag{S17}$$

Thus measurements of $\left.\frac{dm(t)}{dt}\right|_{t=t_{1/2}}$ provide information on how the apparent secondary nucleation rate of α-synuclein varies in the presence of increasing concentrations of β-synuclein. The ThT fluorescence signal was first used to define the change in fibril mass over time, as for the lipid-induced aggregation experiments at pH 6.5, then fitted to a sigmoidal curve and the first derivative at $t_{1/2}$ was computed. This latter term was normalised to the values at [β-synuclein] = 0 μM to give the relative secondary nucleation rate at each β-synuclein:α-synuclein ratio.

Supplementary Figures

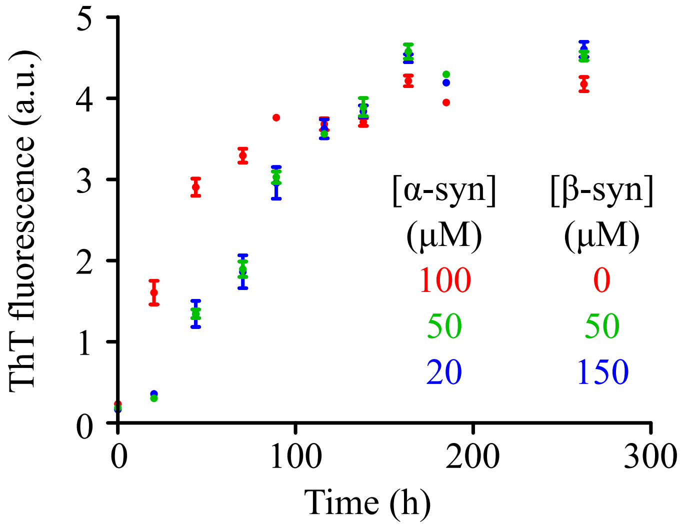

Supplementary Figure 1: Aggregation of α-synuclein in the presence of DMPS vesicles and β-synuclein. Change in ThT fluorescence when α-synuclein was incubated under quiescent conditions in the presence of 350 μM DMPS and increasing concentrations of β-synuclein (100 μM α-synuclein, 0 μM β-synuclein (red); 50 μM α-synuclein, 50 μM β-synuclein (green); and 20 μM α-synuclein, 150 μM β-synuclein (blue)). The conditions used were 30 °C and 20 mM phosphate buffer, pH 6.5.

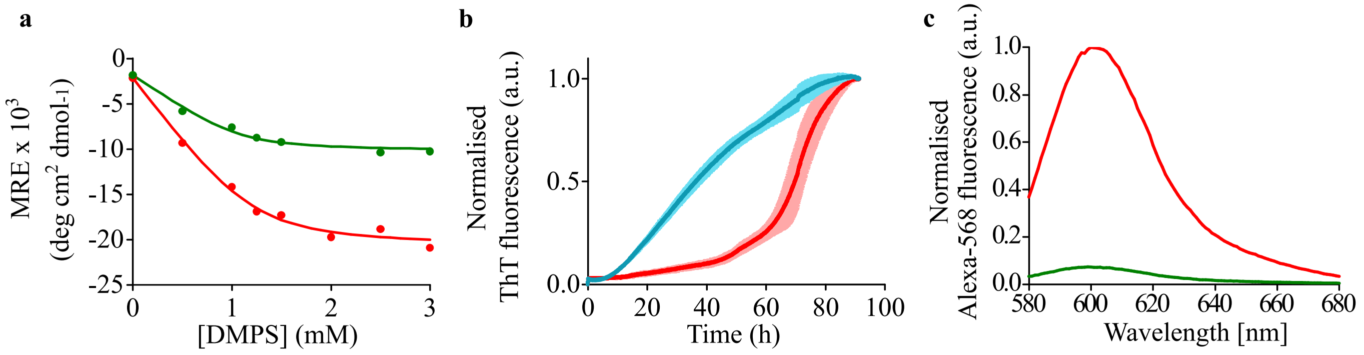

Supplementary Figure 2: Characterisation of the lipid binding and lipid-induced aggregation of Alexa-568-α-synuclein and Alexa-568-β-synuclein in the presence of DMPS. (**a**) Variation of the MRE measured at 222 nm when Alexa-568-α-synuclein (red) and Alexa-568-β-synuclein (green) were incubated in the presence of increasing concentrations of DMPS. Both the curves are well described by a one-step binding model (see Experimental Section for details) to give a K_D of 3.77 $\pm$ 0.61 μM for Alexa-568-α-synuclein and 3.38 $\pm$ 0.83 μM for Alexa-568-β-synuclein. The stoichiometry of binding was 24.6 $\pm$ 1 for Alexa-568-α-synuclein and 21.6 $\pm$ 1.3 for Alexa-568-β-synuclein. (**b**) Change in the ThT fluorescence when 100 μM α-synuclein (blue) or 100 μM Alexa-568-α-synuclein (red) was incubated under quiescent conditions in the presence of 100 μM DMPS vesicles. The quantities of fibrils produced by α-synuclein and Alexa-568-α-synuclein were both 20 $\pm$ 1 μM as measured by absorption spectroscopy (see Experimental Section for details). (**c**) Fluorescence emission spectra (excitation at 568 nm) of resuspended α-synuclein fibrils formed when 100 μM Alexa-568-α-synuclein (red) or 100 μM unlabelled α-synuclein and 100 μM Alexa-568-β-synuclein (green) were incubated in the presence of 350 μM DMPS. The solution conditions for the CD and fluorescence measurements were 20 mM phosphate buffer, pH 6.5 and 30 °C.

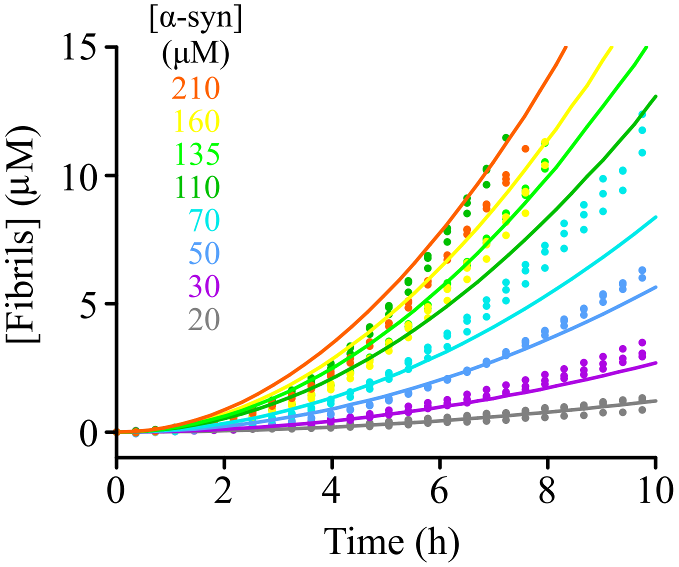

Supplementary Figure 3: Global analysis of the lipid-induced aggregation of α-synuclein in the absence of β-synuclein. The change in the concentration of fibrils formed when α-synuclein was incubated in the presence of 350 μM DMPS (30 °C, pH 6.5) at increasing concentrations of protein as indicated on the figure. The data were fitted globally using AmyloFit to the kinetic model described in the main text and Experimental Section (RMSD=9.80 $\cdot 10^{-7}$).

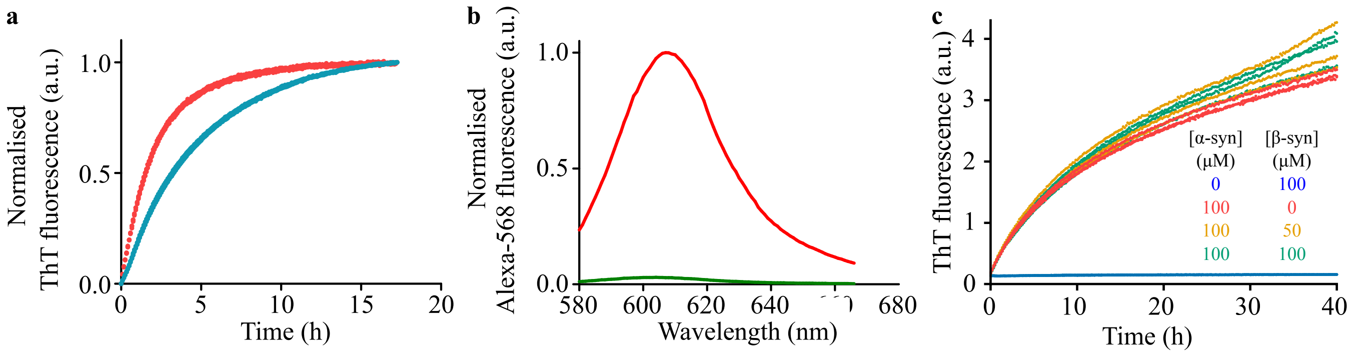

Supplementary Figure 4: Characterisation of the fibrils formed from elongation of α-synuclein seed fibrils in the absence and presence of β-synuclein at pH 6.5. (**a**) Change in the ThT fluorescence when 50 μM unlabelled monomeric α-synuclein (blue) or 50 μM monomeric Alexa-568-α-synuclein (red) was incubated under quiescent conditions in the presence of 5 μM α-synuclein seed fibrils. (**b**) Fluorescence spectra of resuspended α-synuclein fibrils formed when 100 μM monomeric Alexa-568-α-synuclein (red) or 100 μM unlabelled monomeric α-synuclein and 100 μM monomeric Alexa-568-β-synuclein (green) were incubated in the presence of 1 % α-synuclein fibrils. (**c**) Change in the ThT fluorescence signal when solutions of 100 μM monomeric α-synuclein incubated in the presence of 1 μM α-synuclein pre-formed seed fibrils in the absence (red) or the presence of 50 μM (orange) or 100 μM (green) monomeric β-synuclein. The data show that β-synuclein (100 μM) does not detectably elongate α-synuclein seed fibrils (1 μM, blue).

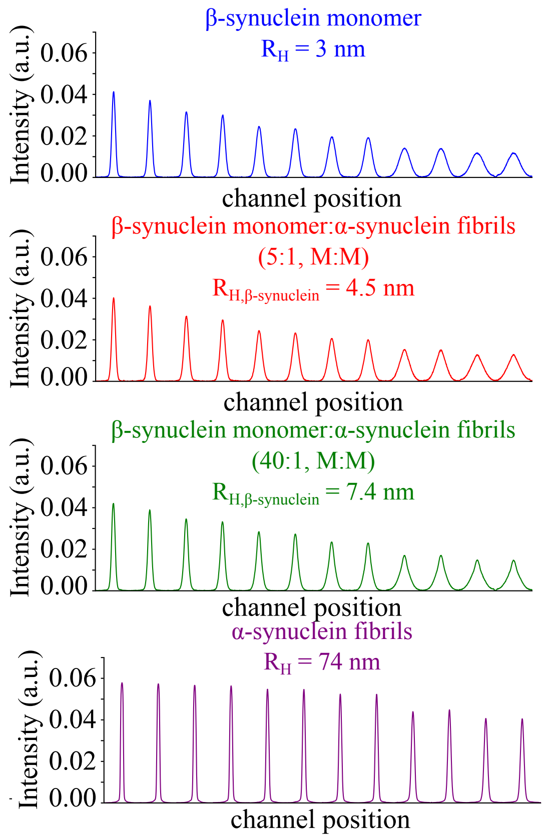

Supplementary Figure 5: The interaction between β-synuclein monomers and α-synuclein fibrils at pH 6.5. Diffusion profiles taken at 12 positions with the microfluidic diffusional sizing device described previously (See Experimental Section for details). The diffusion profiles of 2.5 μM monomeric Alexa-568-β-synuclein (blue) and 100 μM Alexa-647-α-synuclein fibrils (purple) were used to define their respective R_H values. The diffusion profiles of monomeric Alexa-568-β-synuclein in the presence of unlabelled α-synuclein fibrils at concentrations of 12.5 μM (red) and 100 μM (green) were used to estimate the proportion of monomeric β-synuclein attached to α-synuclein fibrils, which was less than 5% and about 10%, for α-synuclein fibrils:β-synuclein monomer ratios of 5 and 40, respectively.

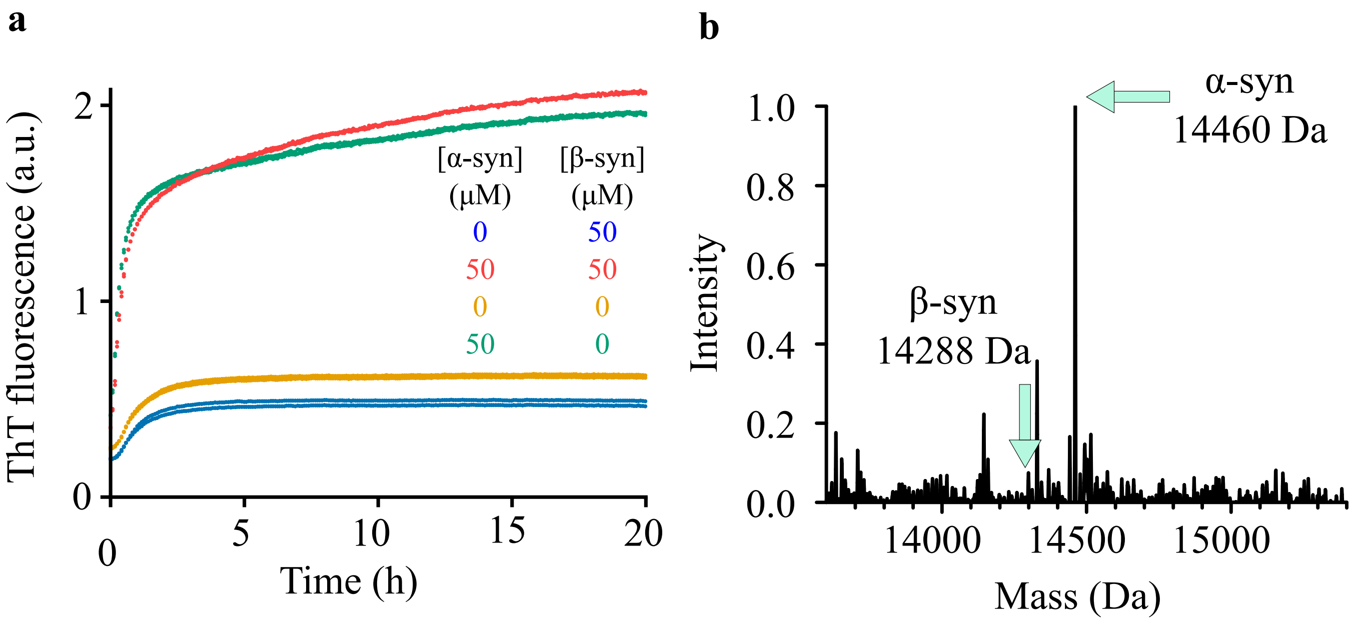

Supplementary Figure 6: The effect of β-synuclein on α-synuclein elongation at pH 4.8. (**a**) Change in ThT fluorescence when 5 μM α-synuclein seed fibrils were incubated under quiescent conditions in the absence of both monomeric α-synuclein and monomeric β-synuclein (yellow), in the presence of 50 μM monomeric β-synuclein (blue) or in the presence of 50 μM monomeric α-synuclein in the absence (green) and presence of 50 μM β-synuclein (red). For yellow and blue traces, the difference in ThT intensity at t = 20 h is very similar to that at t = 0 h (differences at t = 0 h likely due to small differences in the initial seed fibril concentration). (**b**) Mass spectrometric analysis of fibrils formed when 50 nM seed fibrils were incubated in the presence 50 μM monomeric α-synuclein and 50 μM monomeric β-synuclein (red trace on panel A). The data show a peak, at 14,459.74 $\pm$ 2.68, corresponding to the molecular weight of α-synuclein.

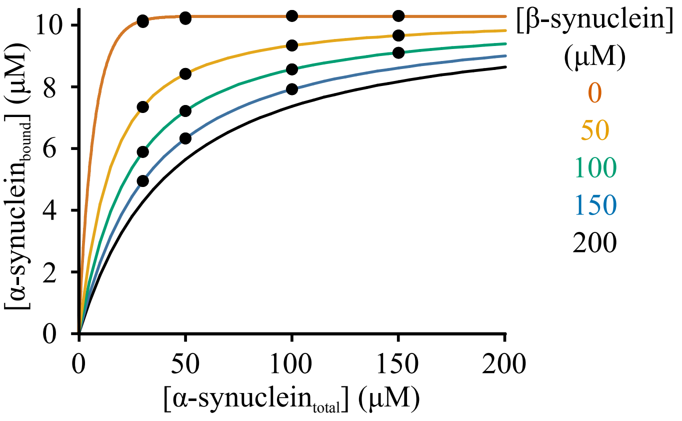

Supplementary Figure 7: Simulation of the variation of the concentration of α-synuclein bound to DMPS with increasing concentration of α-synuclein for different β-synuclein:α-synuclein ratios. For each [β-synuclein] (0 (red), 50 (orange), 100 (green), 150 (blue), 200 (black) μM), the values of the concentration of α-synuclein bound to 350 μM (solid lines) DMPS vesicles was calculated from the competitive binding model (see Experimental Section) using the binding constants (K_D and L) determined using CD measurements individually for α-synuclein and β-synuclein and Eqs. 7 and 8 in the Experimental Section. The black circles represent conditions for which experimental aggregation data were obtained (see Figure 3 in the main text)

References

[1] Arosio, P. *et al.* Microfluidic diffusion analysis of the sizes and interactions of proteins under native solution conditions. *ACS Nano* **10**, 333–341 (2016).

[2] Müller, T. *et al.* Particle-based Monte-Carlo simulations of steady-state mass transport at intermediate Péclet numbers. *Int. J. Nonlinear Sci. Numer. Simul.* **17**, 175–183 (2016).

[3] Buell, A. K. *et al.* Surface attachment of protein fibrils via covalent modification strategies. *J. Phys. Chem. B* **114**, 10925–10938 (2010).

[4] Galvagnion, C. *et al.* Lipid vesicles trigger α-synuclein aggregation by stimulating primary nucleation. *Nat. Chem. Biol.* **11**, 229–234 (2015).

[5] Buell, A. K. *et al.* Solution conditions determine the relative importance of nucleation and growth processes in α-synuclein aggregation. *Proc. Natl. Acad. Sci.* **111**, 7671–7676 (2014).

[6] Meisl, G. *et al.* Molecular mechanisms of protein aggregation from global fitting of kinetic models. *Nat. Protoc.* **11**, 252–272 (2016).

[7] Cohen, S. I. A., Vendruscolo, M., Dobson, C. M. & Knowles, T. P. J. Nucleated polymerization with secondary pathways. II. Determination of self-consistent solutions to growth processes described by non-linear master equations. *J. Chem. Phys.* **135**, 065106 (2011).
